# Recurrent Recruitment Manoeuvres Improve Lung Mechanics and Minimize Lung Injury during Mechanical Ventilation of Healthy Mice

**DOI:** 10.1371/journal.pone.0024527

**Published:** 2011-09-15

**Authors:** Lucy Kathleen Reiss, Anke Kowallik, Stefan Uhlig

**Affiliations:** Institute of Pharmacology and Toxicology, Medical Faculty of RWTH Aachen University, Aachen, Germany; Ludwig-Maximilians-Universität München, Germany

## Abstract

**Introduction:**

Mechanical ventilation (MV) of mice is increasingly required in experimental studies, but the conditions that allow stable ventilation of mice over several hours have not yet been fully defined. In addition, most previous studies documented vital parameters and lung mechanics only incompletely. The aim of the present study was to establish experimental conditions that keep these parameters within their physiological range over a period of 6 h. For this purpose, we also examined the effects of frequent short recruitment manoeuvres (RM) in healthy mice.

**Methods:**

Mice were ventilated at low tidal volume V_T_ = 8 mL/kg or high tidal volume V_T_ = 16 mL/kg and a positive end-expiratory pressure (PEEP) of 2 or 6 cmH_2_O. RM were performed every 5 min, 60 min or not at all. Lung mechanics were followed by the forced oscillation technique. Blood pressure (BP), electrocardiogram (ECG), heart frequency (HF), oxygen saturation and body temperature were monitored. Blood gases, neutrophil-recruitment, microvascular permeability and pro-inflammatory cytokines in bronchoalveolar lavage (BAL) and blood serum as well as histopathology of the lung were examined.

**Results:**

MV with repetitive RM every 5 min resulted in stable respiratory mechanics. Ventilation without RM worsened lung mechanics due to alveolar collapse, leading to impaired gas exchange. HF and BP were affected by anaesthesia, but not by ventilation. Microvascular permeability was highest in atelectatic lungs, whereas neutrophil-recruitment and structural changes were strongest in lungs ventilated with high tidal volume. The cytokines IL-6 and KC, but neither TNF nor IP-10, were elevated in the BAL and serum of all ventilated mice and were reduced by recurrent RM. Lung mechanics, oxygenation and pulmonary inflammation were improved by increased PEEP.

**Conclusions:**

Recurrent RM maintain lung mechanics in their physiological range during low tidal volume ventilation of healthy mice by preventing atelectasis and reduce the development of pulmonary inflammation.

## Introduction

Mechanical ventilation (MV) of mice is increasingly used in biomedical research. While the mechanisms of ventilator-induced lung injury (VILI) have been explored intensively [1], experimental conditions required to keep physiological parameters stable in mice during ventilation for several hours are not well defined. Major reasons for this are the focus on the mechanisms of VILI and the lack of comprehensive monitoring of pulmonary and cardiovascular key parameters in most studies.

Monitoring of key physiological parameters is standard during mechanical ventilation of humans and should be aimed also in experimental research. These key parameters need to reflect both the pulmonary (e.g. tidal volume, airway pressure) and the cardiovascular (e.g. heart rate, blood pressure) consequences of MV as well as oxygenation and acid-base status. Although MV may affect all these parameters, these entities have rarely been assessed together in the same study in mice (Table 1). Table 1 summarizes ventilation studies, in which either lung impedance was measured or ventilation was performed for at least four hours. The table reveals that many studies that have focused on lung mechanics examined only a relatively short period of ventilation [2]–[5] and only one study fulfilled both inclusion criteria [6]. Interestingly, studies in which mice were ventilated for more than three hours often provided cardiovascular parameters, but neglected the examination of lung functions [7]–[10]. Some studies even completely lacked physiological parameters, although in several cases the authors referred to preliminary experiments that were not included in the published data [11]–[14]. We believe that, without comprehensive information on physiological parameters, it is difficult to properly assess, standardize and compare the various ventilation strategies.

**Table 1:** Comparison of mouse ventilation models.

Reference	Experimental design				Monitored parameters			
	Ventilation	V_T_ [ml/kg]	PEEP [cmH_2_O]	RM	Lung functions	BGA	BP/cardiac activity	SpO_2_
[2]	30 min	7/10	2/0	*	+	−	−/P	+
[3]	60 min	8	6/3	**	+	−	−	−
[4]	140 min	30/10	2/0	2x/min	+	+	−/ECG	−
[5]	150 min	8	6/2	1x/5 min or 1x/75 min	+	−	−/P	+
[60]	4 h	25/7	−	−	+	−	−/ECG	−
[12]	4 h	20/6	2	−	−	−	−	−
[61]	4 h	30	−	−	−	−	−	−
[62]	4 h	20/7	0−2	−	−	−	−	−
[7]	4 h	8	4	−	−	+	+/−	−
[11]	5 h	30/6	−	−	−	−	−/−	−
[8]	5 h	15/7.5	2	−	−	+	+/+	−
[63]	6 h	24	−	−	−	−	+/P	−
[9]	6 h	12	6	*	−	+	+/P	+
[64]	4 h/8 h	20/10	2	1x/h	−	−	+/−	−
[10]	8 h	12	2	−	−	+	+/−	−
Present study	6 h	16/8	2	1x/5 min or 1x/60 min or no RM	+	+	+/ECG, P	+

The table lists those ventilation studies that analyzed lung functions by measurement of lung impedance and studies in which mice were ventilated for at least four hours. V_T_: tidal volume, PEEP: positive end-expiratory pressure, RM: recruitment manoeuvre, BGA: blood gas analysis, BP: blood pressure, P: pulse, ECG: electrocardiogram, SpO_2_: pulse oximetry. * One RM at the beginning of ventilation. ** Two RMs at the beginning of ventilation.

Studies on the mechanisms of VILI have identified several beneficial ventilation strategies, among them low tidal volume (V_T_) ventilation and application of recruitment manoeuvres (RM) as well as high positive end-expiratory pressure (PEEP). Although RM have a sound physiological basis, it remains unclear how they should be applied [15]. In principal, RM may be used to reopen atelectatic lung areas in injured lungs or to prevent atelectasis in healthy lungs. The latter application requires lower recruitment pressures and hence can be applied more frequently. Without RM, pulmonary compliance is likely to decrease as shown already many years ago in anesthetized dogs both during spontaneous breathing and during mechanical ventilation [16]. In addition, regular short recruitment manoeuvres have proved to be useful in models of isolated rat and mouse lungs [17], [18]. In mechanically ventilated mice the usefulness of RM with the specific aim to keep pulmonary compliance and other lung functions constant has been explored only sporadically. Previous RM studies in healthy mice have focused on short periods of ventilation and potential lung injury, but did not compare ventilation with and without RM over several hours [4], [5]. In addition, the effect of RM on blood pressure in mice is not well defined, although increased intrathoracic pressure might decrease cardiac output [19]. The beneficial effects of PEEP have been demonstrated in several animal studies [20]–[22]. PEEP helps to prevent repetitive alveolar collapse and preserves surfactant function [1], [23]. While it is widely accepted that adequate PEEP together with low V_T_ is a protective ventilation strategy, the effects of recurrent RM in this setting are unknown.

The present study had several aims: (1) In order to assess the consequences of MV properly, we established a set-up that permits the ventilation of mice under permanent monitoring of clinically relevant physiological parameters. (2) We used this set of parameters to define ventilatory conditions that guarantee stable lung mechanics, hemodynamics, acid base status and oxygenation over six hours. In particular, we studied the input impedance of the lung at low frequencies to distinguish mechanical properties of conductive airways and the distal lung [24]. (3) We investigated the physiological effects of recurrent RM on lung mechanics at two different tidal volumes (8 vs. 16 mL/kg) and at two different PEEP-levels (2 vs. 6 cmH_2_O). (4) Since inflammatory lung injury may occur even with ‘non-injurious’ ventilation [7], [8], [25], we also assessed the extent of pulmonary inflammation, i.e. microvascular permeability, pulmonary sequestration of leukocytes, production of pro-inflammatory cytokines and lung histopathology. Our findings in healthy animals show that low V_T_ ventilation will only maintain lung functions and gas exchange in a normal range if recurrent RM are used. In addition, recurrent RM reduced the extent of pulmonary inflammation, although mild inflammation was present in the lungs of all ventilated animals. The higher PEEP of 6 cmH_2_O was beneficial regarding lung mechanics, oxygenation and pulmonary inflammation. Thus, we suggest that a preferable ventilation strategy is one that combines low tidal volumes with sizable PEEP and recurrent recruitment manoeuvres.

## Methods

### Mice

Experiments were performed with female C57BL/6 N mice (Charles River, Sulzfeld, Germany) aged 8 to 12 weeks, weighing 20 to 25 grams. The experimental protocols were in accordance with the German animal protection law and approved by regional governmental authorities (Landesamt für Natur, Umwelt und Verbraucherschutz NRW, permission number: AZ 8.87-50.10.35.085).

### Surgical procedures, mechanical ventilation and physiological monitoring

Mice were initially anaesthetized with an intraperitoneal injection of pentobarbital sodium [75 mg/kg] and fentanyl [40 µg/kg]. Anaesthesia was maintained with pentobarbital sodium [20 mg/kg] via an intraperitoneal catheter every 30 to 60 minutes. Mice were tracheotomized with a 20-gauge cannula and connected to the ventilator. A catheter was inserted into the carotid artery, which allowed blood pressure monitoring and permanent infusion of 0.9% NaCl (200 µL/h) to prevent hypovolaemia and thrombus formation. Pulsoxymetry was performed with a tail clip (MouseOx, STARR Life-Science, Oakmont, PA, USA). Blood pressure and ECG were recorded permanently (PowerLab, ADInstruments, Spenbach, Germany). Heart rate was calculated from the ECG. Body temperature was measured rectally and kept stable between 36.5°C and 37.5°C by a homeothermic blanket (Harvard Apparatus Holliston, MA, USA).

Mice were ventilated for six hours with the flexiVent ventilator (SCIREQ, Montreal, Canada). All mice survived the protocol. Mice were either ventilated with low tidal volume (lowV_T_) of 8 mL/kg and a frequency of 180 min^−1^ or high tidal volume (highV_T_) of 16 mL/kg and a frequency of 90 min^−1^, so that both groups received the same minute volume. The highV_T_ group was ventilated with 3% CO_2_ to maintain normocapnia without further decreasing ventilation frequency. The fraction of inspired oxygen (FiO_2_) was 0.5 in all experiments. A positive end-expiratory pressure (PEEP) of either 2 cmH_2_O or 6 cmH_2_O was applied. One recruitment manoeuvre (RM) with 30 cmH_2_O pressure and six seconds duration was performed after onset of ventilation to open airspaces and standardize lung volume. Resistance, compliance and impedance of the lung were measured by the low-frequency forced oscillation technique every ten minutes.

**Figure 1:**
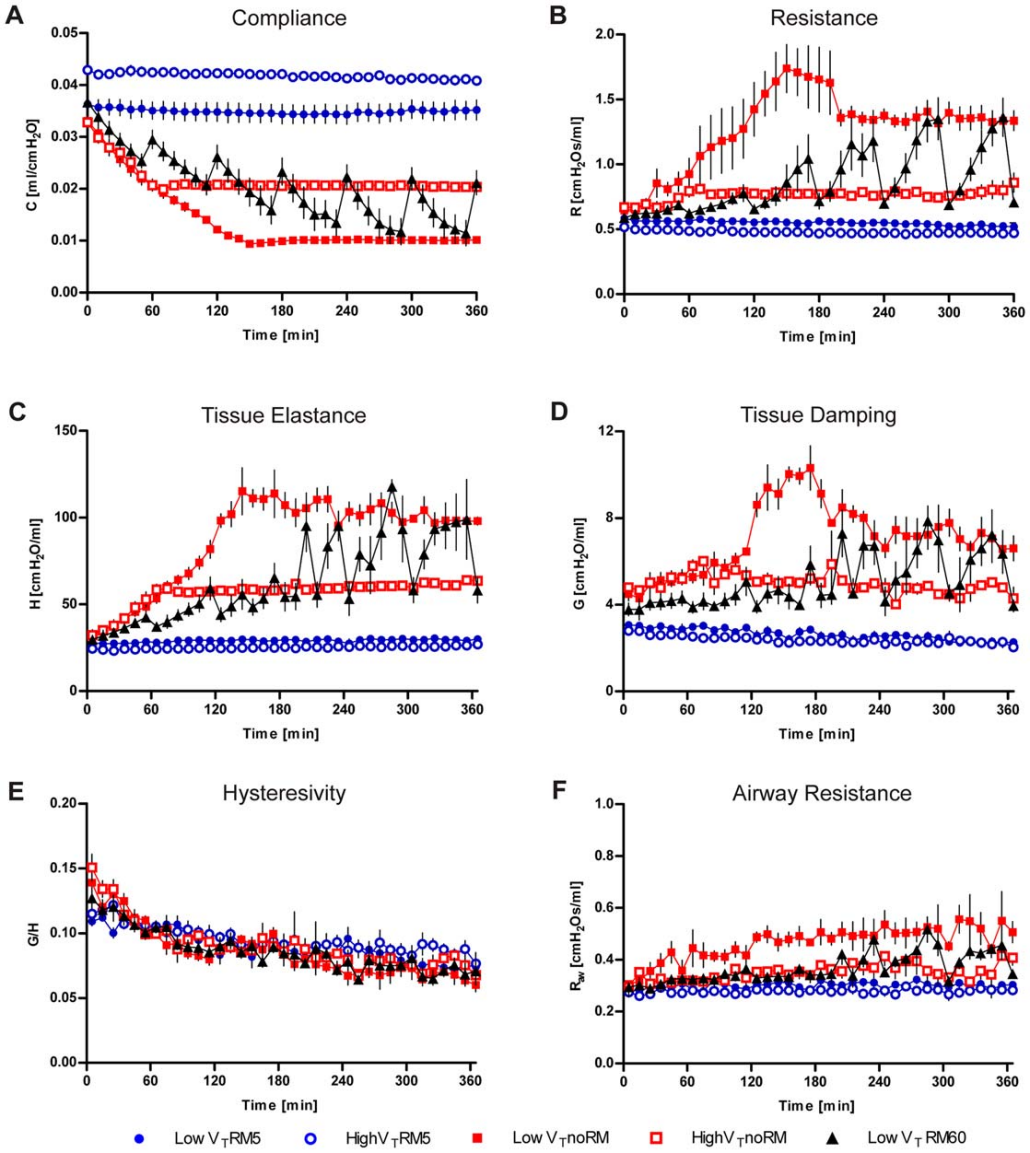
Lung mechanics. Mice were ventilated for six hours with low V_T_  = 8 mL/kg or high V_T_  = 16 mL/kg, PEEP  = 2 cmH_2_O and RM every five minutes (RM5), every 60 minutes (RM60) or without RM (noRM). Lung mechanics were measured every ten minutes by the forced oscillation technique. (LowV_T_RM5: n = 6, highV_T_RM5: n = 6, lowV_T_noRM: n = 5, highV_T_noRM: n = 4, lowV_T_RM60: n = 5). P-values for group comparisons are shown in supplementary Table S1.

**Figure 2:**
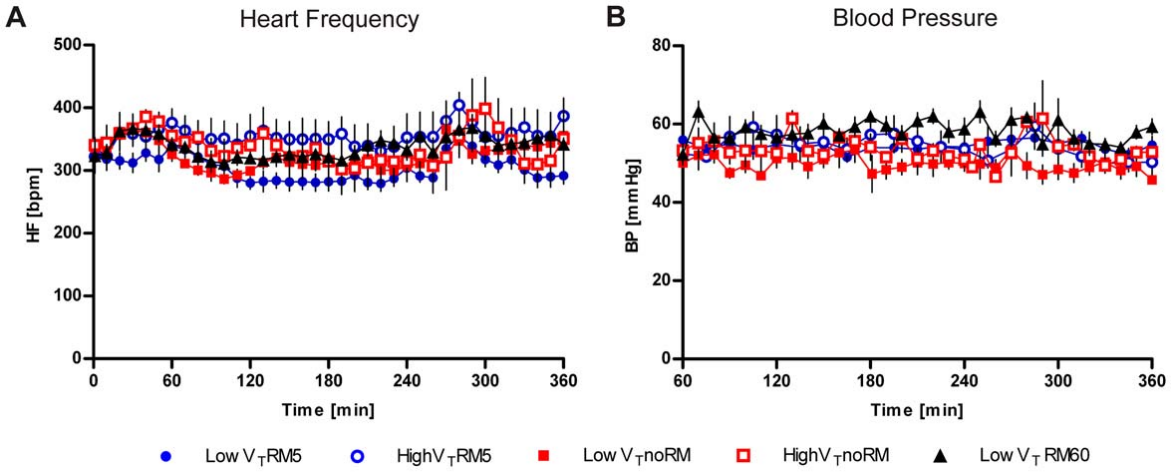
Heart rate and blood pressure. Electrocardiogram (ECG) was recorded permanently. **A**. Heart frequency (HF) was calculated simultaneously from the ECG and is displayed in beats per minute (bpm). **B**. Mean arterial blood pressure (BP) was measured via a catheter in the carotid artery.(LowV_T_RM5 n = 6, highV_T_RM5 n = 6, lowV_T_noRM n = 5, highV_T_noRM n = 4, lowV_T_RM60 n = 5).

Two different series of experiments were performed and analyzed separately (summarized in Table 2). The first series of experiments (Fig. 1–2 3 4 5 6 7 8) was performed with a PEEP of 2 cmH_2_O. Repeated recruitment manoeuvres of 1 s duration and 30 cmH_2_O peak pressure were applied every five minutes (RM5) in one lowV_T_ (lowV_T_RM5) and one highV_T_ group (highV_T_RM5) or every 60 minutes (RM60) in one lowV_T_ group (lowV_T_RM60). One lowV_T_ and one highV_T_ group were ventilated without RM (lowV_T_noRM and highV_T_noRM). One group of anaesthetized but not ventilated mice served as control. A second series of experiments (Fig. 9–10 11 12) was performed, in which mice were ventilated with a PEEP of 6 cmH_2_O and low V_T_. RM were performed every five minutes (PEEP6_RM5), every 60 minutes (PEEP6_RM60) or not at all (PEEP6_noRM).

**Figure 3:**
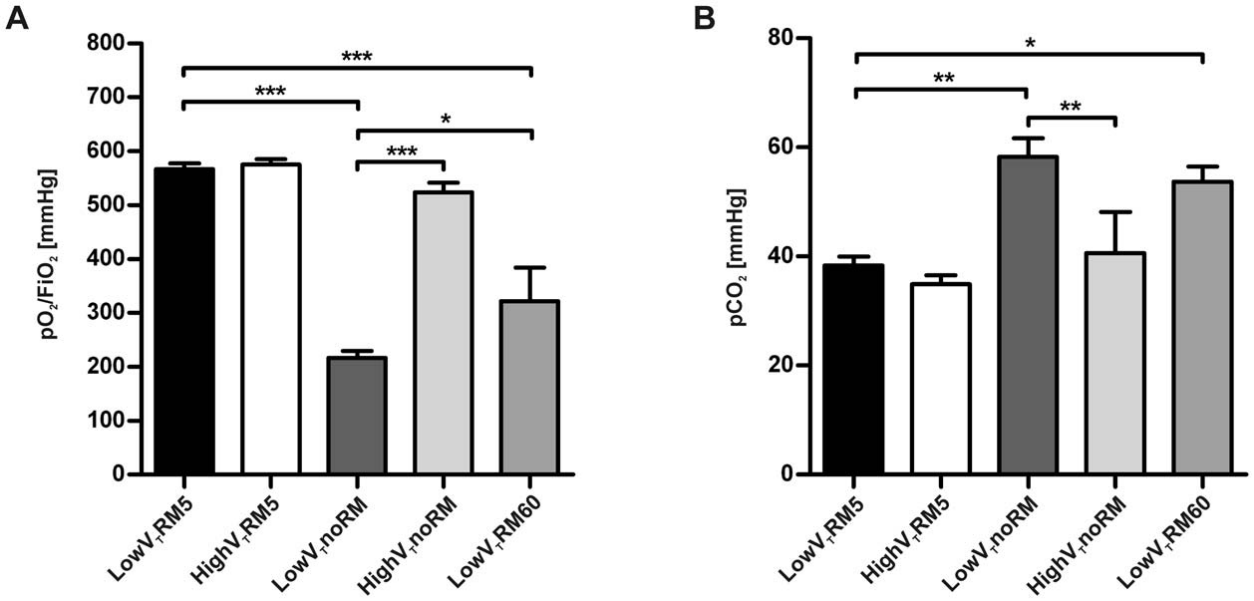
Blood gas results. Arterial blood was analysed after six hours of ventilation. **A**. pO_2_/FiO_2_ ratio was calculated, FiO_2_ was 0.5. **B**. Comparison of pCO_2_ levels. (LowV_T_RM5 n = 6, highV_T_RM5 n = 6, lowV_T_noRM n = 5, highV_T_noRM n = 4, lowV_T_RM60 n = 5). * p<0.05, ** p<0.01, *** p<0.001.

**Figure 4:**
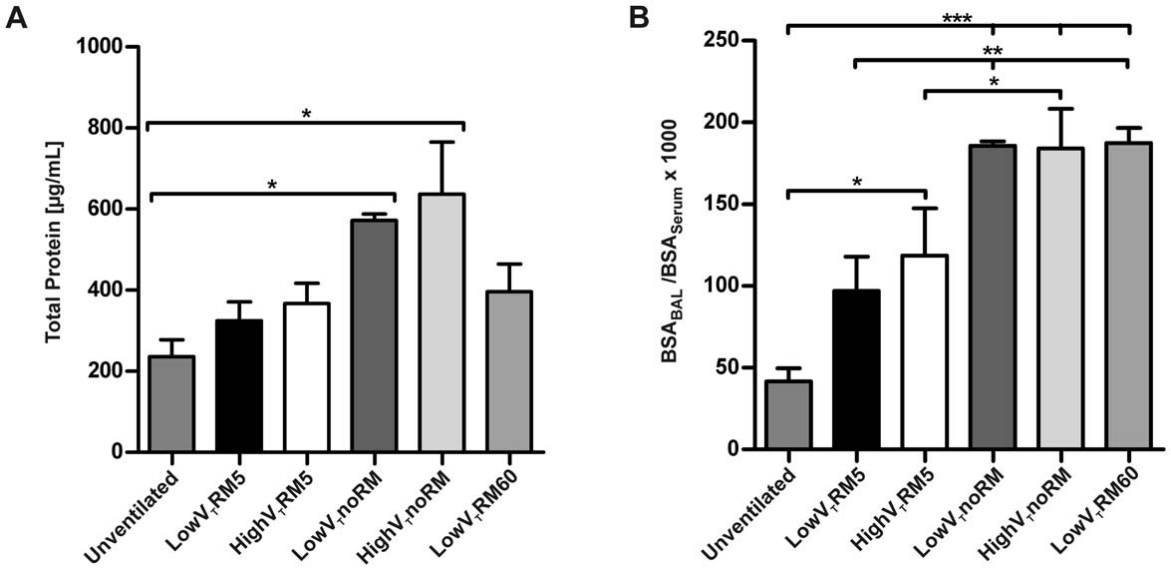
Protein leakage. **A**. Total protein levels were measured with DC protein assay. (Unventilated n = 5, lowV_T_RM5 n = 6, highV_T_RM5 n = 6, lowV_T_noRM n = 4, highV_T_noRM n = 4, lowV_T_RM60 n = 5). **B**. BSA was quantified in BAL and serum by ELISA and the ratio was calculated. (Unventilated n = 5, lowV_T_RM5 n = 5, highV_T_RM5 n = 5, lowV_T_noRM n = 4, highV_T_noRM n = 4, lowV_T_RM60 n = 5). * p<0.05, ** p<0.01, *** p<0.001.

**Figure 5:**
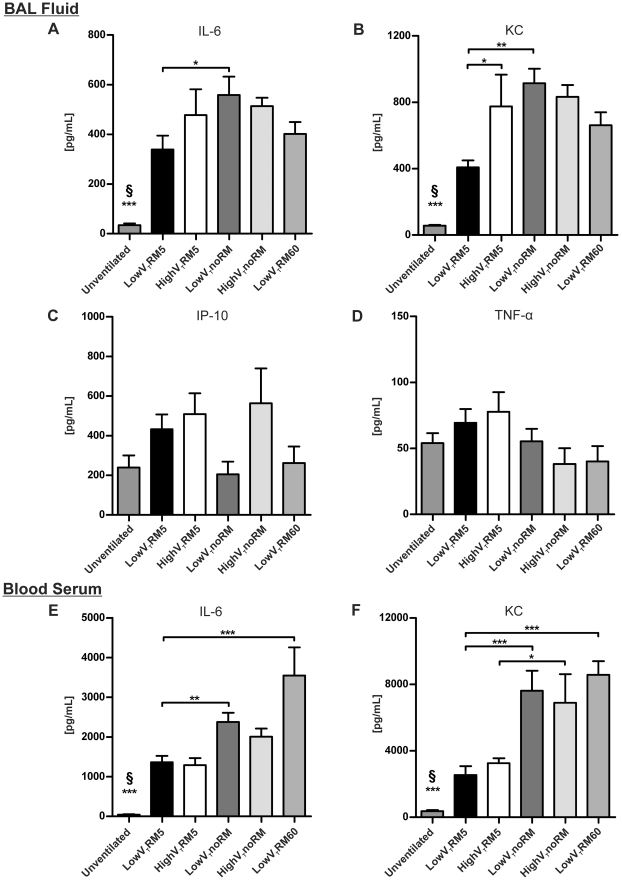
Cytokine levels. Cytokines were quantified in blood serum or BAL supernatant with commercial ELISA kits after six hours of ventilation and in unventilated mice. (Unventilated: n = 5, lowV_T_RM5 n = 6, highV_T_RM5 n = 6, lowV_T_noRM n = 4 in BAL and n = 5 in serum, highV_T_noRM n = 4, lowV_T_RM60 n = 5). * p<0.05, ** p<0.01, *** p<0.001, § *** p<0.001 versus all other groups).

**Figure 6:**
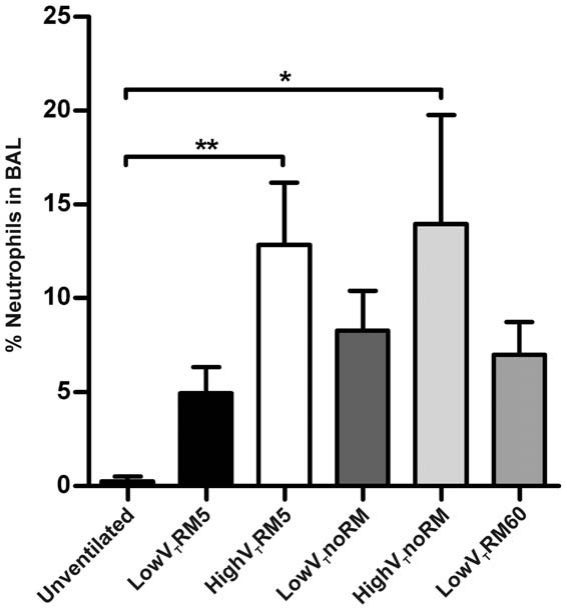
Neutrophils in BAL fluid. BAL fluid was subjected to cytospin preparation, followed by Diff-Quick staining. From each preparation 400 cells were counted and percentage of neutrophils was calculated. (Unventilated: n = 5, lowV_T_RM5 n = 5, highV_T_RM5 n = 5, lowV_T_noRM n = 4, highV_T_noRM n = 4, lowV_T_RM60 n = 5). § *** p<0.001 versus all other groups.

**Figure 7 pone-0024527-g007:**
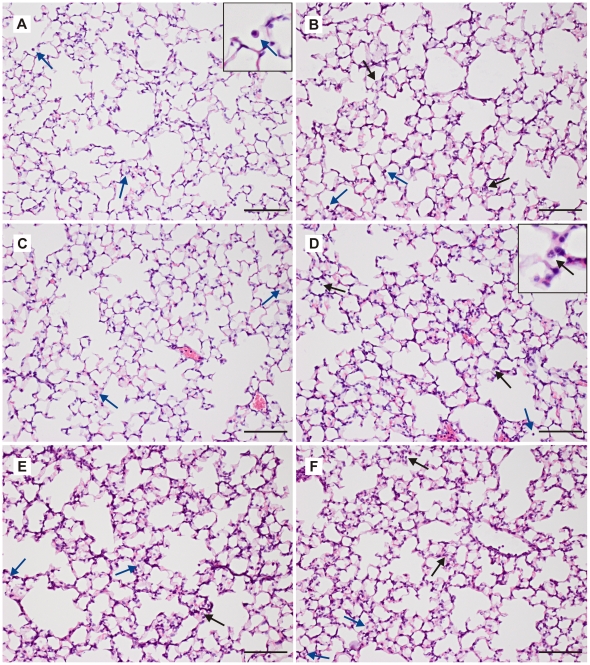
Lung histopathology. Representative lung sections stained with hematoxylin and eosin from: **A**. unventilated control, **B**. lowV_T_RM60, **C**. lowV_T_RM5, **D**. highV_T_RM5, **E**. lowV_T_noRM and **F**. highV_T_noRM mice. Black arrows indicate neutrophils; blue arrows indicate intra-alveolar monocytes and macrophages, scale bars 100 µm; magnification 200x. Insets in A and D contain enlarged images of exemplary leukocytes.

**Figure 8:**
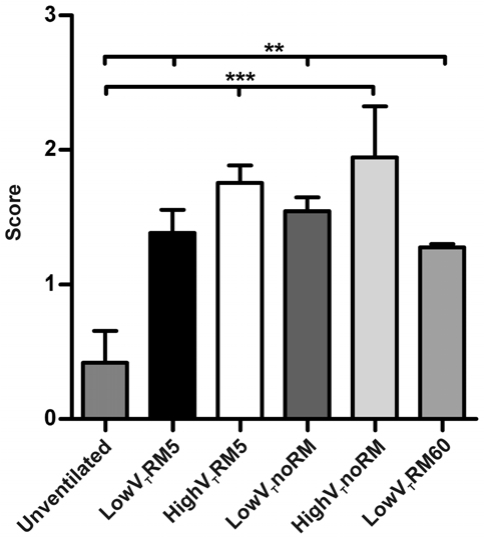
Histopathological scoring. Scoring criteria were: neutrophils in the alveolar or interstitial space, alveolar septal thickening, alveolar congestion and formation of hyaline membranes. Scores from 0 to 4 were given according to the number of fulfilled criteria. (Unventilated: n = 5, lowV_T_RM5 n = 5, highV_T_RM5 n = 5, lowV_T_noRM n = 4, highV_T_noRM n = 4, lowV_T_RM60 n = 5). ** p<0.01, *** p<0.001.

**Figure 9:**
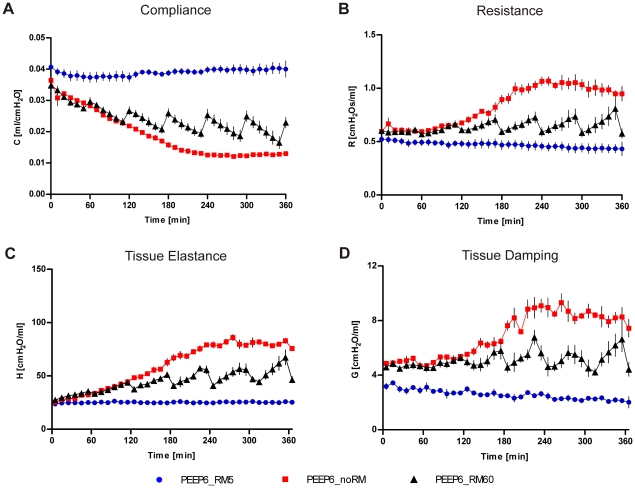
Lung mechanics with 6 cmH_2_O PEEP. Mice were ventilated for six hours with low V_T_  =  8 mL/kg, PEEP  =  6 cmH_2_O and RM every five minutes (RM5), every 60 minutes (RM60) or without RM (noRM). Lung mechanics were measured with the forced oscillation technique every ten minutes. (n = 4 in all groups). Please see supplementary Table S2 for p-values of group comparisons.

**Figure 10:**
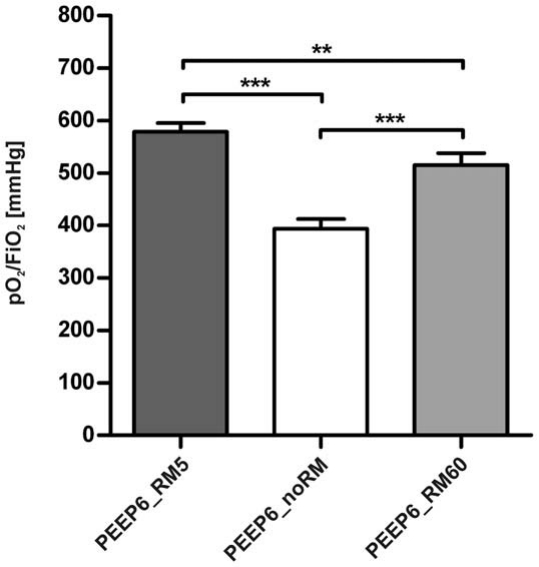
Oxygenation with 6 cmH_2_O PEEP. Arterial blood was analysed after six hours of ventilation and the pO_2_/FiO_2_ ratio was calculated. (n = 4 in all groups). ** p<0.01, *** p<0.001.

**Figure 11:**
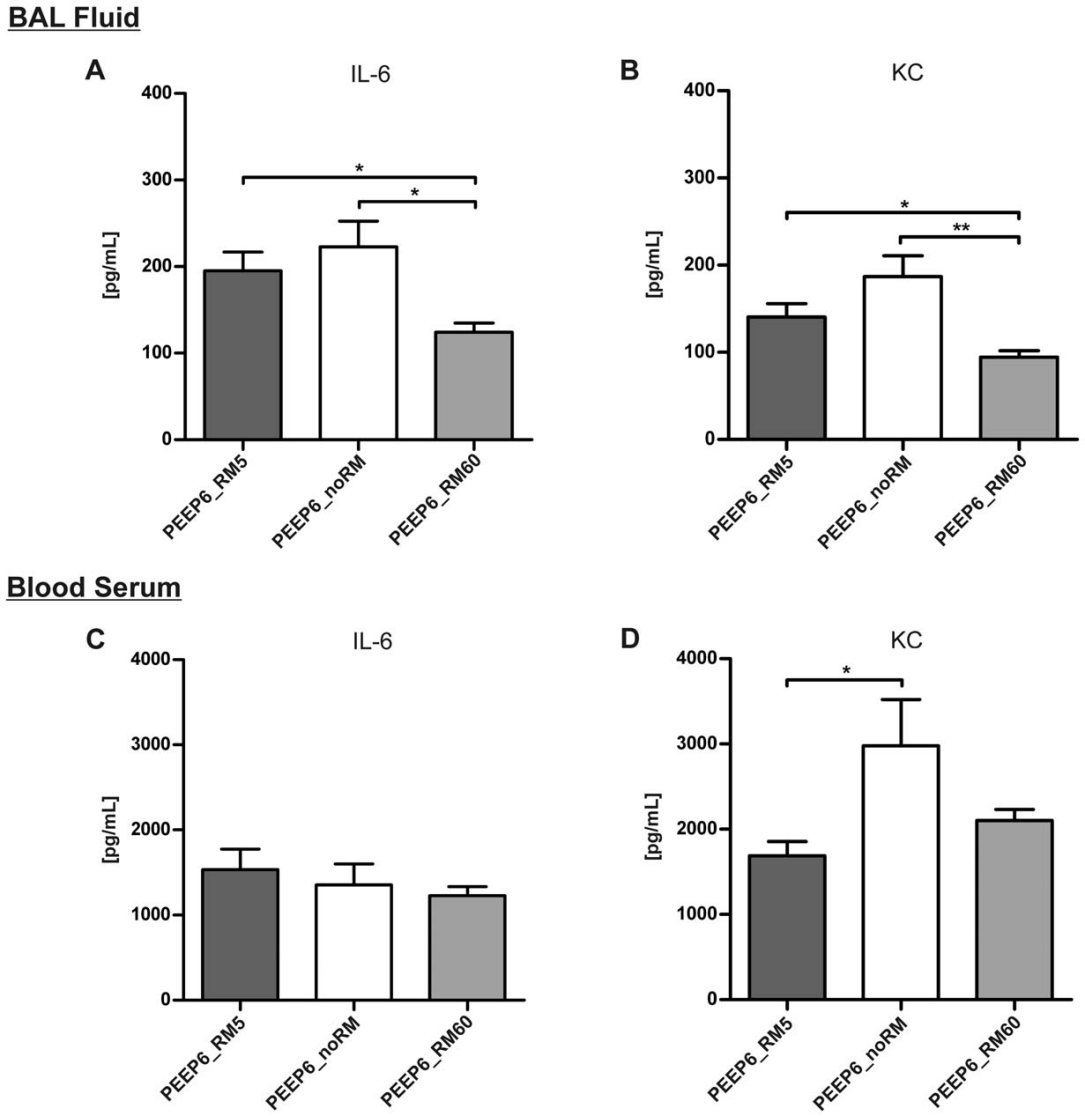
Cytokine levels with 6 cmH_2_O PEEP. Cytokines levels after six hours of ventilation were measured in blood serum or BAL supernatant by ELISA. (n = 4 in all groups). * p<0.05, ** p<0.01.

**Figure 12:**
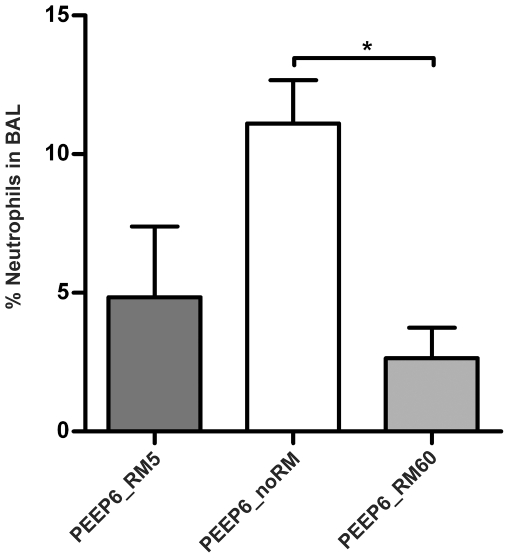
Neutrophils in BAL fluid with 6 cmH_2_O PEEP. Leukocytes were counted after cytospin preparation of BAL fluid. From each preparation 400 cells were counted and percentage of neutrophils was calculated. (n = 4 in all groups). * p<0.05.

Mice received 1 mg bovine serum albumin (BSA) intravenously 90 min before exsanguination for analysis of microvascular permeability. Mice were sacrificed by exsanguination via the carotid artery. Blood samples from ventilated mice were analysed for pO_2_, pCO_2_, pH, HCO_3_^−^ and standard base excess (SBE) by blood gas analysis (ABL700, Radiometer, Copenhagen, Denmark). Blood gas analysis from anaesthetized control mice was not representative due to reduced breathing activity.

**Table 2:** Experimental groups.

	First experimental series					Second experimental series		
	LowV_T_ RM5	HighV_T_ RM5	LowV_T_ noRM	HighV_T_ noRM	LowV_T_ RM60	PEEP6 _RM5	PEEP6 _noRM	PEEP6 _RM60
**V_T_ [ml/kg]**	8	16	8	16	8	8	8	8
**f [min^−1^]**	180	90	180	90	180	180	180	180
**PEEP [cmH_2_O]**	2	2	2	2	2	6	6	6
**RM/h**	12	12	-	-	1	12	-	1
**FiO_2_**	0.5	0.5	0.5	0.5	0.5	0.5	0.5	0.5
**CO_2_ [%]**	0	3	0	3	0	0	0	0

V_T_: tidal volume, f: breathing frequency, PEEP: positive end-expiratory pressure, RM: recruitment manoeuvre of 1 s and 30 cmH_2_O pressure, FiO_2_: fraction of inspired oxygen, lowV_T_: low tidal volume, highV_T_: high tidal volume, RM5: one recruitment manoeuvre every five minutes, noRM: no recruitment manoeuvres, RM60: one recruitment manoeuvre every 60 minutes, PEEP6: PEEP = 6 cmH_2_O.

### Right lung: lung permeability, cytokine detection, differential cell count

After thoracotomy lungs were perfused free of blood with ice cold phosphate buffered saline. Bronchoalveolar lavage of the right lung was performed by instilling two times 300 µL NaCl via tracheal tubing. About 500 µL BAL fluid was retrieved from each mouse. BAL fluid was centrifuged and the supernatant was frozen for quantification of proteins. The pellet was transferred to a cytospin preparation, followed by a modified Giemsa stain (Diff-Quick; Medion Diagnostics, Düdingen, CH) and differential leukocyte count. Cytokine levels in serum and BAL fluid were quantified with commercial enzyme-linked immunosorbent assays (ELISA) (R+D Systems, Abingdon; UK). BSA was quantified in blood serum and BAL fluid by ELISA (Bethyl Laboratories, Montgomery, USA). The ratio of BSA in serum and BAL fluid was calculated to determine microvascular permeability. Additionally, total protein levels were measured using a DC protein assay (BioRad, Hercules, CA, USA).

### Left lung: lung histopathology

The left lung was filled with 4% formalin for fixation and embedded in paraffin for histopathological examination. Sections of 3 µm thickness were stained with hematoxilin and eosin (HE). Histopathology was evaluated in a blinded manner. The following scoring system based on four criteria was used for each histological section: neutrophils in the alveolar or interstitial space, alveolar septal thickening, alveolar congestion and formation of hyaline membranes. Each criterion scored one point, if present, thus scores ranged from 0 (none criterion found) to 4 (all four criteria observed). Nine to ten sections per lung were evaluated and the mean was calculated for every lung and every group.

### Statistical analysis

Lung mechanics were analysed with the mixed model procedure followed by correction for false discovery rate (FDR). To evaluate lung mechanics from the groups lowV_T_RM60 and PEEP6_RM60 in a linear model, one time point every 60 minutes before and one after RM were analyzed separately, resulting in two separate linear slopes (lowV_T_RM60a and PEEP6_RM60a: before RM, lowV_T_RM60b and PEEP6_RM60b: after RM). BoxCox transformation was performed to achieve homoscedasticity and normal distribution, when necessary. Analyses of parametric data were carried out with One-Way Analysis of Variance and FDR correction. Non-parametric data were analysed with the Kruskall-Wallis test followed by Dunn's post-test. Data in figures are shown as mean ± standard error of the mean (SEM). P-values<0.05 were considered as significant. Statistical analyses were carried out with JMP 7 or SAS 9.1 software (SAS Institute Inc., Cary, NC, USA).

## Results

### A. First series of experiments: PEEP  = 2 cmH_2_O

#### Lung mechanics

The first series of experiments was performed at a PEEP  = 2 cmH_2_O, a PEEP level that is commonly used in animal studies that aim to study VILI (see Table 1). Ventilation with 8 mL/kg and no RM (lowV_T_noRM) caused a continuous decrease of pulmonary compliance (C) over more than two hours that reached a plateau at approximately 30% of the physiological baseline value (Fig. 1). Within this time pulmonary resistance (R) nearly tripled. Resistance and compliance showed less dramatic trends in mice ventilated with 16 mL/kg and no RM (highV_T_noRM). In this group C plateaued earlier (60 min) at about 60% of the baseline value and R increased to a lesser degree (∼25%). R and C differed significantly between the lowV_T_noRM and the highV_T_noRM group (R: p<0.01; C: p<0.001). Respiratory input impedance revealed major alterations in the periphery of the lung. Tissue damping (G) and tissue elastance (H) were elevated in both highV_T_noRM and lowV_T_noRM mice, reaching significantly higher values in the lowV_T_noRM group (G: p<0.01; H: p<0.001). In the latter group, airway resistance (R_aw_) was increased as well. The increase in G, H and R_aw_ indicates that low V_T_ ventilation without RM leads to closure of peripheral areas of the lung and causes a small degree of airway constriction. In consequence, hysteresivity (G/H  =  η) decreased slightly over time, but was in a comparable range in all groups.

These findings indicate that ventilation without RM leads to impaired lung functions, which stabilize within two hours, though at a low level. We therefore examined the effect of repetitive RM (30 cmH_2_O) every 5 min. In both the lowV_T_RM5 and the highV_T_RM5 group, lung mechanics stayed in a physiological range and remained unchanged during the whole experiment. Depending on the different tidal volumes, C and H differed reciprocally between low- and highV_T_ groups (p<0.05). The stability of the lung mechanics demonstrates that application of deep inflations of 1 s duration and 30 cmH_2_O are sufficient to prevent the deterioration of lung functions in healthy mouse lungs ventilated with 2 cmH_2_O PEEP and moderate tidal volumes.

Further, we examined whether it would suffice to apply RM only every 60 min instead of every 5 min. This was studied in the low V_T_ group (lowV_T_RM60) only. In this group, respiratory conditions were instable. R and C worsened comparable to the lowV_T_noRM group, but improved significantly after each RM. These alterations were reflected in changes in G and H. However, even though each RM was beneficial, after six hours tissue elastance had increased permanently, indicating that one deep inflation per hour is not sufficient to maintain lung volume at base line values. This is illustrated by the finding that H (p<0.001), R, C, G (all p<0.01) and R_aw_ (p<0.05) were significantly different between the lowV_T_RM60 and the lowV_T_RM5 group.

#### Heart frequency, blood pressure and oxygenation

Anaesthesia with pentobarbital sodium resulted in reduced heart frequency (300–400 min^−1^) and mean blood pressure (50–60 mmHg), compared to average data of unsedated mice. ECG (data not shown), HF and BP remained unchanged throughout ventilation and were not significantly different between the groups (Fig. 2). This demonstrates that ventilation and recruitment strategy did not affect the circulation. The fluid support with 200L NaCl per hour in all groups was adequate to keep blood pressure stable. Oxygen saturation, measured by pulsoxymetry, was over 90% in all mice at all times (data not shown).

#### Blood gas analysis

Blood gas analyses revealed that infusion of saline and application of 3% CO_2_ in the high V_T_ groups were adequate to keep the acid-base status stable and that ventilation resulted in normocapnia in the lowV_T_RM5 and highV_T_RM5 group, with pCO_2_ levels ranging from 35 to 40 mmHg (Table 3). The pO_2_/FiO_2_ ratio was between 530 and 620 mmHg in both RM5 groups and in the highV_T_noRM group (Figure 3). Most mice ventilated with highV_T_noRM had physiological pCO_2_ levels after six hours. The pO_2_/FiO_2_ ratio was decreased significantly to around 200 mmHg in the lowV_T_noRM group, indicating acute respiratory failure. In this group, impaired gas exchange was further indicated by an increase in pCO_2_ levels to about 60 mmHg, leading to respiratory acidosis with a mean pH of 7.22. Mice in the lowV_T_RM60 group showed very heterogeneous pO_2_/FiO_2_ ratios between 260 and 570 mmHg and markedly increased pCO_2_ levels as well as initiating hypercapnic acidosis. Bicarbonate (HCO_3_^−^) was only slightly reduced, resulting in negative values for standard base excess, but did not indicate metabolic imbalance.

**Table 3:** Blood gas results.

	LowV_T_RM5	HighV_T_RM5	LowV_T_noRM	HighV_T_noRM	LowV_T_RM60
**pO_2_ [mmHg]**	283.5±13.4	287.7±12.7	108.6±13.6	261.8±17.8	205.5±72.4
**pCO_2_ [mmHg]**	38.4±3.9	34.9±4.0	58.3±7.61	40.6±15.1	50.1±9.4
**pH**	7.33±0.03	7.36±0.04	7.22±0.03	7.32±0.08	7.28±0.05
**HCO_3_^- ^[mmol/L]**	19.5±1.6	19.2±1.6	23.6±2.3	19.7±3.4	21.8±1.9
**SBE [mmol/L]**	− 6.0±1.6	− 5.5±1.7	− 3.5±2.1	− 5.4±2.3	− 3.5±1.6

Blood gas analyses from arterial blood after six hours of mechanical ventilation with PEEP  = 2 cmH_2_O. Data are shown as mean ± standard deviation. SBE: standard base excess, lowV_T_: low tidal volume, highV_T_: high tidal volume, RM5: one recruitment manoeuvre every five minutes, noRM: no recruitment manoeuvres, RM60: one recruitment manoeuvre every 60 minutes.

#### Pulmonary microvascular permeability

Total protein levels were increased in all ventilated mice, compared to unventilated controls. Protein levels were highest in the groups without RM (Fig. 4A). The BSA BAL/serum ratio confirmed that ventilation augmented microvascular permeability in the lung, particularly when inspiratory pressures were high, as in the groups ventilated without repetitive recruitment (Fig. 4B).

#### Pro-inflammatory cytokine levels

Interleukin-6 (IL-6) and Keratinocyte-derived chemokine (KC) were elevated in the BAL fluid and blood serum from all ventilated mice (Fig. 5A, B, E, F). BAL fluid from mice ventilated with lowV_T_RM5 showed a less dramatic increase in these pro-inflammatory cytokines. In both RM5 groups serum levels of IL-6 and KC were clearly lower than in all other ventilation groups. Interestingly, Interferon-γ induced protein (IP-10) and Tumour necrosis factor-α (TNF-α) were not significantly increased in the BAL from ventilated mice, compared to unventilated controls (Fig. 5C, D).

#### Differential cell count in the BAL fluid

The only cell types found in BAL fluid of unventilated control mice were monocytes and macrophages. In contrast, all samples from ventilated mice also contained neutrophils (Fig. 6). Numbers of neutrophils were highest in mice ventilated with high V_T_. Considerably less neutrophils were recruited to alveoli of lowV_T_ mice, whereof the group lowV_T_RM5 had the lowest neutrophil count.

#### Histopathology

Histopathological samples revealed that no severe lung injury was induced by the ventilation strategies applied (Fig. 7). Nonetheless, significant histopathological alterations were present in all ventilated lungs and were most obvious in mice ventilated with high V_T_ (Fig. 8). Alveolar septal thickening was observed in most of the ventilated lungs, resulting in a score of one. A score of two was given, when neutrophils were observed additionally, as in most lungs from highV_T_ mice. This is in line with the results from the differential cell count in the BAL fluid. Only some highV_T_noRM mice showed alveolar congestion, bringing the lung injury score up to three. Hyaline membranes were not observed in any sections, indicating that the applied ventilation strategies induced only moderate injury in the healthy lungs.

### B. Second series of experiments: PEEP  = 6 cmH_2_O

#### Lung mechanics

In the second series of experiments the ventilation strategies RM5, noRM and RM60 were applied to mice ventilated with low V_T_ and a PEEP of 6cmH_2_O, to find out whether the impairment of lung mechanics during ventilation with noRM and RM60 could be prevented by a higher PEEP (Fig. 9). Ventilation with RM5 at a PEEP of 6 cmH_2_O (PEEP6_RM5) showed stable lung functions. Ventilation without RM (PEEP6_noRM) resulted, notwithstanding the increased PEEP, in a strong decrease in C during the first 180 min of ventilation until a plateau was reached at about 30% of the initial C. Correspondingly R, G and H increased and finally doubled in this group. Also ventilation with RM60 (PEEP6_RM60) did not suffice to keep lung functions entirely stable at a PEEP of 6 cmH_2_O. Although the decrease in C between the RMs was relatively moderate, the initial C was not maintained over six hours. R, in contrast, returned to the initial value after each RM. In line with this, lung impedance measurements revealed a moderate increase in H, but not in G after six hours. All three groups differed significantly from each other regarding C, R, H and G, except from the parameters measured in the PEEP6_RM60 group directly after the RM. R_aw_ and hysteresivity were not different between the groups and R_aw_ remained at baseline in all experiments (data not shown). These results indicate that an elevation of PEEP, although it helps to stabilize lung mechanics, is not sufficient to replace repetitive RM.

#### Blood gas analysis

One aim of the second series of experiments was to investigate whether a higher PEEP can prevent the worsening of gas exchange in mice ventilated at a PEEP  = 2 cmH_2_O and RM60 or noRM. The pO_2_/FiO_2_ ratio was around 500 mmHg in the PEEP6_RM60 group and around 400 mmHg in the PEEP6_noRM group (Fig. 10). Both groups differed significantly from the control group PEEP6_RM5, which showed unimpaired gas exchange with a pO_2_/FiO_2_ ratio of about 600 mmHg This further demonstrates that regular RMs are necessary even with a PEEP of 6 cmH_2_O. In accordance, pCO_2_ levels were significantly elevated in the groups PEEP6_RM60 and PEEP6_noRM, compared to the control group (data not shown).

#### Pro-inflammatory cytokine levels

In the second set of experiments IL-6 and KC levels in the BAL fluid were highest in the PEEP6_noRM group, followed by the PEEP6_RM5 group (Fig. 11A, B). Both mediators were lowest in the PEEP6_noRM group. IL-6 in the blood serum was not significantly different between these three groups (Fig. 11C). KC levels were highest in the group ventilated without RM, but differed only slightly between the groups ventilated with RM5 and RM60 Fig. 11D). These results indicate that a higher PEEP helps preventing the release of pro-inflammatory mediators induced by formation of atelectasis.

#### Differential cell count in the BAL fluid

In the second part of the study, in which all mice were ventilated with low V_T_, neutrophil numbers were clearly highest in the group PEEP = 6_noRM. Numbers of neutrophils were lower in the BAL fluid from PEEP6_RM5 mice and lowest in the PEEP6_RM60 group (Fig. 12).

## Discussion

There is an increasing demand for mechanical ventilation of mice, not only in pulmonary research, but also in many other areas such as neurology [26], imaging [27] or cardiovascular research [28]. Monitoring of pulmonary and vital parameters is an essential prerequisite for correct interpretation of data from such models. The present study indicates that the general notion according to which low tidal volumes and sizable PEEP levels are preferable is – at least in healthy mice – only true when repetitive recruitment manoeuvres are applied.

The effects of repetitive RM are not well established neither in experimental animals nor in men, one reason being that trials are difficult to compare [19]. To date, application of RM in patients with healthy lungs has been explored only sporadically [29], although there is some evidence that RM may support protective ventilation by improving oxygenation and lung mechanics, thereby allowing reduction of V_T_ [30], [31]. Clinically, it has been shown that low V_T_ ventilation improves the outcome of patients with injured lungs [32], and also reduces inflammation in those with healthy lungs [33]. Taken together, it seems highly important to gain more information on the effects of repetitive ‘sigh-like’ recruitment manoeuvres during low V_T_ ventilation.

We demonstrated that application of deep inflations of 1 s duration and 30 cmH_2_O peak pressure in five minute intervals was sufficient to keep respiratory mechanics stable and thereby lung volume constant in low and high V_T_ ventilation, without increasing PEEP over 2 cmH_2_O (Fig. 1). We initially selected a relatively low PEEP level, because we plan to use the same PEEP in future studies on the molecular mechanisms of VILI, where high PEEP levels are known to be protective (many VILI models use no PEEP at all). Since RMs can be performed with varying duration, peak pressure and end-expiratory pressure [34], we chose a very short type of RM, which we considered to be non-injurious to the lung and not to impair cardiac output. Accordingly, blood pressure and heart frequency were stable, regardless of the ventilation strategy. Although blood pressure was in a low range due to anaesthesia with pentobarbital, so that small changes might have been masked, this did not result in reflex tachycardia or impairment of microcirculation, as the pH values indicate (Table 3). These stable cardiovascular conditions were also guaranteed by several supportive measures such as constant fluid support (200 µL/h), regular administration of pentobarbital via an intraperitoneal catheter, and an automated system to keep the body temperature constant. Further, it can be speculated that the low blood pressure is protective in this model, since edema formation is generally aggravated by high blood pressure.

In order to keep blood gases stable, the low and high V_T_ groups had to be ventilated with different respiratory rates and, based on other studies [35], we cannot completely rule out that this might have affected the outcome of our study. Our conditions were chosen to provide the same minute volume at both tidal volumes. The supplementation of 3% CO_2_ to the inhaled gas mixture in the high V_T_ groups was adequate to prevent hypocapnia, which otherwise would have occurred at a respiratory rate of 90 min^−1^ in the highV_T_RM5 group. 3% CO_2_ alone did not induce hypercapnia (see Table 3). Thus, a protective effect of hypercapnia during MV, which is described in several studies [36], [37], seems unlikely.

A pressure of 30 cmH_2_O for each RM was necessary to maintain lung volume stable. In accordance with previous studies [4], [5] RM with 25 cmH_2_O peak pressure were not sufficient to avoid a loss of lung volume (data not shown). The double sigmoidal nature of the murine pressure volume curve [38] may explain the positive response to RM with 30 cmH_2_O pressure. According to Zosky et al. [38], pressures above 20 cmH_2_O induce fundamental changes in the murine lung, resulting in increased compliance. These changes are proposed to be either due to reopening of collapsed alveoli or to a secondary population of alveoli that are present but not aerated below a critical transrespiratory pressure [39]. In the present study, measurement of lung input impedance revealed that the alterations in pulmonary resistance and compliance in RM60 and noRM mice were predominantly due to changes in the periphery of the lung. Although we observed significant differences in R_aw_ between RM5, RM60 and noRM mice ventilated at the same V_T_, this effect was rather small, indicating that bronchoconstriction of large airways is of minor importance to explain our findings. The strong increase in G and H in mice ventilated without RM demonstrates a progressive loss in lung volume [24]. Increases in tissue damping reflect energy dissipation and are associated with an increase in tissue resistance and/or regional heterogeneity as result of peripheral airway constriction, whereas tissue elastance reflects energy storage in lung tissues and represents lung stiffness [5]. The small decrease in hysteresivity shows that H increased slightly stronger than G. Further, it underlines that impaired lung functions were not primarily due to heterogeneity caused by narrowing of small airways [40]. Thus narrowing of conductive airways played only a minor role in this model and we conclude that the loss of lung volume was mainly due to formation of atelectasis.

In fact, lung volume declined only to a certain threshold and a plateau phase was reached in all mice ventilated without RM. This supports the hypothesis that depending on the pressure applied to the lung some populations of alveoli are open and others closed [38]. The minimal lung volumes in the plateau phase differed according to the employed tidal volume, leading to the highest tissue resistance and elastance values in the lowV_T_noRM group. Formation of atelectasis was more severe in low V_T_ than in high V_T_ ventilation, as shown elsewhere [3], [4], indicating that low V_T_ ventilation without RM was injurious. These findings are in line with a previous work showing that low V_T_ ventilation augmented lung injury in isolated rat lungs, proposing that the degree of lung injury is dependent on the end-expiratory lung volume [41]. Ventilation with high V_T_ resulted in a higher compliance and thereby to some degree protected from the decline in lung volume. However, the application of one RM per hour was not sufficient to keep tissue resistance and elastance entirely stable over six hours, demonstrating that the beneficial effect of recruitment is transient and formation of atelectasis is reversible only to a certain point. The present data emphasize the importance of frequent deep inflations in mice in order to maintain lung functions in a physiological range.

Further evidence for the formation of atelectasis was given by blood gas analysis, which revealed impaired gas exchange in lowV_T_noRM and lowV_T_RM60 mice (Fig. 3, Table 3). The present study demonstrates that impairment of gas exchange, to a level that would fulfil the clinical definition for ARDS [42], can be caused by a ventilation strategy that leads to derecruitment of lung volume. On the other hand, our data on lung histopathology, neutrophil count and BAL levels of pro-inflammatory cytokines indicate that inflammatory lung injury was not severe even in the lowV_T_noRM group. Notably, neutrophil counts and histopathological alterations (Fig. 6–8) did not correlate well with gas exchange and lung functions, further supporting our conclusion that atelectasis rather than inflammation was the major reason for the impaired gas exchange in the groups ventilated without sufficient RM. In fact, neutrophil infiltration was highest in the high V_T_ groups, suggesting ventilation-induced inflammation (biotrauma) as a mechanism.

Nonetheless, protein leakage, indicating increased microvascular permeability, another hallmark of VILI, was highest in those low V_T_ groups that developed atelectasis due to lacking adequate RM (noRM, RM60) (Fig. 4). Collapse of lung units is accompanied by an increase in vascular filtration pressure, leading to edema formation, when vascular barrier functions are impaired [43]. This is in line with the observed alveolar septal thickening. Furthermore, microvascular leakage may also interfere with pulmonary surfactant, thus contributing to a reduction of compliance [44], which was clearly present in this model. Finally, in atelectatic lungs shear forces between closed and open alveoli may develop [1]. Taken together we conclude that a lack of recruitment manoeuvres may cause what has been termed atelectotrauma.

The present study demonstrates that repetitive recruitment manoeuvres are not only beneficial for lung functions, gas exchange and barrier function, but also reduce the liberation of pro-inflammatory cytokines (Fig. 5). This is an important finding, because in theory frequent deep inspirations might be a trigger for stretch-induced pro-inflammatory mediator release. IL-6 and KC were increased in the BAL and blood serum of all ventilated mice and it appears that ventilation will always cause mild inflammation in the lungs, both experimentally [7], [8], [25] and clinically [45], [46]. The underlying mechanisms may be related to the surgery or mechanotransductive processes, i.e. stretch-activation of signalling cascades, or to increased vascular shear stress in those groups ventilated with higher positive pressures [47]. IL-6 and KC were significantly lower in the BAL fluid of lowV_T_RM5 mice compared to all other groups, demonstrating that this ventilation strategy was the most protective one. It is well-established that protective ventilation, minimizing overdistension and recruitment/derecruitment of the lung, attenuates cytokine liberation in the lung and circulation [48]. Interestingly, the blood serum of both V_T_ groups ventilated with RM5 contained significantly lower levels of IL-6 and KC than the corresponding groups ventilated with RM60 or noRM. This shows that the RM5 procedure was also protective at higher tidal volumes. The high levels of IL-6 and KC in the BAL of highV_T_RM5 mice indicate that the inflammatory response was initiated in the lung. Notably, neither levels of IP-10 nor of TNF-α - two cytokines that are frequently found in severe forms of acute lung injury - were altered significantly in ventilated mice, compared to unventilated controls. The relatively high baseline values for TNF- α in unventilated mice are most likely due to the fact that BAL samples had to be diluted on order to gain enough volume for the assay. Concentrations were calculated by multiplication with the dilution factor, possibly leading to higher concentrations than actually present in the BAL. Nevertheless, all groups were analyzed at the same time and cytokine levels were in the same range in all groups, allowing a comparison. IP-10 is proposed to play a role in development of ARDS and is further considered as a useful biomarker for lung disease [49], [50]. TNF-α is considered as a mediator of pulmonary inflammation released by injurious ventilation [51], [52]. The finding that these mediators remained unaltered by ventilation further suggests that lung injury was only moderate in the current model.

This study aimed to define settings for ventilation under stable conditions and therefore a second set of experiments was performed with a PEEP of 6 cmH_2_O, to address the question whether a higher PEEP could prevent the severe impairment of lung functions and the effects on oxygenation and inflammation, which were observed in the lowV_T_noRM and lowV_T_RM60 groups ventilated with a PEEP of 2 cmH_2_O. Lung mechanics and blood gas results revealed that a higher PEEP, even when combined with RM60, was not sufficient to prevent formation of atelectasis. C decreased and R, G and H increased strongly during the first 180 min of the experiments in the PEEP6_noRM group before parameters reached a plateau. A previous study demonstrated that an increase in PEEP from 2 cmH_2_O to 6 cmH_2_O alone did not prevent an increase in R_aw_, G and H during 150 min of ventilation, but was more effective during pressure controlled RM [5]. The present study shows that a combination of higher PEEP and short RM every 60 minutes does not suffice to prevent impairment of lung functions and that frequent RM are required, as with a PEEP of 2 cmH_2_O. Of note, the increase in R_aw_, which was observed in the groups lowV_T_noRM and lowV_T_RM60 ventilated with a PEEP  = 2 cmH_2_O, was completely abolished in all groups ventilated with a PEEP  = 6 cmH_2_O, indicating that a higher PEEP helps to prevent narrowing of large airways during MV.

It is well known that PEEP plays a critical role in preventing/reducing lung injury [23]. The combination of low V_T_ and high PEEP has already proved to be beneficial for the outcome of patients with ARDS [53]. Further, an experimental ventilation strategy using low V_T_, PEEP and RM resulted in a better outcome in ARDS patient than a strategy without RM, although differences were not significant due to limitations of the trial [54]. Given the significance of biotrauma [55], [56], it is an important question whether a ventilation strategy that combines high PEEP with RM attenuates pulmonary inflammation during low V_T_. The second part of this work showed that differences in cytokine levels between mice ventilated with the same RM strategies were smaller with 6 cmH_2_O than with 2 cmH_2_O PEEP. IL-6 serum levels were even in a comparable range in all PEEP6 groups. Interestingly, the highest IL-6 and KC BAL levels were detected in the PEEP6_noRM group. This correlates well with the neutrophil count that was also highest in this group. Thus, the application of RM appears to be beneficial with respect to pulmonary inflammation also at a PEEP of 6 cmH_2_O.

### Conclusions

We compared lung mechanics during ventilation with and without RM in healthy mice over six hours during close monitoring of many clinically relevant parameters including lung mechanics, cardiovascular functions and gas exchange. Stable cardiovascular conditions resulted amongst others from appropriate fluid support, emphasizing the need for careful monitoring and stabilisation of vital parameters in animal studies.

The present study shows that protective non-injurious ventilation requires the application of frequent non-injurious recruitment manoeuvres and low V_T_. A ventilation pattern including deep inflations is closer to the variable breathing pattern of spontaneously breathing subjects than monotonic MV without variation in V_T_ or breathing frequency, supporting the finding that variable or ‘noisy’ ventilation improves protective ventilation in the porcine and human lung [57], [58]. Beyond that, variable ventilation, characterised by breath-to-breath variation of V_T_ and breathing frequency, has been shown to be beneficial in terms of reducing VILI in mice with injured lungs [59].

Furthermore, our data indicate that a PEEP level of 6 cmH_2_O has protective effects and is therefore advisable in studies that require MV. A low PEEP of 2 cmH_2_O combined with recurrent RM is less protective, but still suffices to gain stable respiratory conditions and may be preferable in models that aim to investigate VILI.

We conclude that ventilation with low V_T_, recurrent RM and sizable PEEP is the most protective ventilation strategy for healthy mice.

## Supporting Information

Table S1 **P-values for group comparisons of lung mechanics wit 2 cmH_2_O PEEP.**
(DOC)

Table S2 **P-values for group comparisons of lung mechanics with 6 cmH_2_O PEEP.**
(DOC)
