# Supplementary material for: Recurrent Recruitment Manoeuvres Improve Lung Mechanics and Minimize Lung Injury during Mechanical Ventilation of Healthy Mice
Source: PLoS One. 2011 Sep 15;6(9):e24527. doi: 10.1371/journal.pone.0024527 (PMC3174196; doi:10.1371/journal.pone.0024527)
Supplement: Table S1 — P-values for group comparisons of lung mechanics wit 2 cmH2O PEEP. (DOC) [file pone.0024527.s001.doc]

| **Table S1. P-values for group comparisons of lung mechanics wit 2 cmH_2_O PEEP** | | | | | |
| --- | --- | --- | --- | --- | --- |
|  | **C** | **R** | **G** | **H** | **R_aw_** |
| **LowV_T_RM5 – LowV_T_noRM** | 0.001 | 0.001 | 0.05 | 0.001 | 0.001 |
| **LowV_T_RM5 – HighV_T_RM5** | n.s. | 0.05 | n.s. | n.s. | 0.05 |
| **LowV_T_RM5 – LowV_T_RM60a** | 0.001 | 0.001 | 0.01 | 0.001 | 0.001 |
| **LowV_T_RM5 – LowV_T_RM60b** | 0.01 | 0.01 | 0.05 | 0.01 | 0.001 |
| **HighV_T_RM5 – HighV_T_noRM** | 0.001 | 0.001 | 0.05 | 0.001 | 0.001 |
| **LowV_T_noRM – LowV_T_RM60b** | 0.01 | 0.01 | 0.05 | 0.001 | 0.001 |
| **LowV_T_noRM – HighV_T_noRM** | 0.01 | 0.001 | n.s. | 0.01 | 0.001 |
| **LowV_T_RM60a – LowV_T_RM60b** | 0.01 | 0.05 | 0.05 | 0.05 | 0.01 |
| C: compliance, R: resistance, G: tissue damping, H: tissue elastance, R_aw_: airway resistance. p < 0.05 was considered as statistically significant, n.s. not significant. | | | | | |
